# Emerging Evidence that ApoC-III Inhibitors Provide Novel Options to Reduce the Residual CVD

**DOI:** 10.1007/s11883-019-0791-9

**Published:** 2019-05-20

**Authors:** Marja-Riitta Taskinen, Chris J. Packard, Jan Borén

**Affiliations:** 1 Research Programs Unit, Diabetes and Obesity, University of Helsinki, Helsinki, Finland; 2 Institute of Cardiovascular and Medical Sciences, University of Glasgow, Glasgow, UK; 3 Wallenberg Laboratory, Department of Molecular and Clinical Medicine, University of Gothenburg and Sahlgrenska University Hospital, Gothenburg, Sweden

**Keywords:** ApoC-III, Lipoproteins, Triglycerides, Remnants, CVD, genetic variants

## Abstract

**Purpose of Review:**

Apolipoprotein C-III (apoC-III) is known to inhibit lipoprotein lipase (LPL) and function as an important regulator of triglyceride metabolism. In addition, apoC-III has also more recently been identified as an important risk factor for cardiovascular disease. This review summarizes the mechanisms by which apoC-III induces hypertriglyceridemia and promotes atherogenesis, as well as the findings from recent clinical trials using novel strategies for lowering apoC-III.

**Recent Findings:**

Genetic studies have identified subjects with heterozygote loss-of-function (LOF) mutations in APOC3, the gene coding for apoC-III. Clinical characterization of these individuals shows that the LOF variants associate with a low-risk lipoprotein profile, in particular reduced plasma triglycerides. Recent results also show that complete deficiency of apoC-III is not a lethal mutation and is associated with very rapid lipolysis of plasma triglyceride-rich lipoproteins (TRL). Ongoing trials based on emerging gene-silencing technologies show that intervention markedly lowers apoC-III levels and, consequently, plasma triglyceride. Unexpectedly, the evidence points to apoC-III not only inhibiting LPL activity but also suppressing removal of TRLs by LPL-independent pathways.

**Summary:**

Available data clearly show that apoC-III is an important cardiovascular risk factor and that lifelong deficiency of apoC-III is cardioprotective. Novel therapies have been developed, and results from recent clinical trials indicate that effective reduction of plasma triglycerides by inhibition of apoC-III might be a promising strategy in management of severe hypertriglyceridemia and, more generally, a novel approach to CHD prevention in those with elevated plasma triglyceride.

## Introduction

Apolipoprotein C-III (apoC-III), a small protein (79 amino acid residues) that contains two amphipathic helices [1], was discovered almost 50 years ago but until it was recognized as an important risk factor for cardiovascular disease (CVD) did not attract much attention. It resides on circulating lipoproteins including high-density lipoproteins (HDL), low-density lipoprotein (LDL), and triglyceride-rich lipoproteins (TRLs) such as chylomicrons (CM) and very low density lipoprotein (VLDL). Today, we know that apoC-III is a multifaceted protein with major physiological relevance. It not only regulates triglyceride metabolism but also is believed to participate in pathological processes involved in atherosclerosis.

### Role of ApoC-III Biology in the Human Pathophysiology of CVD

The structure and function of apoC-III have been studied for many years but we still do not have a clear picture of its many interactions with lipoprotein particles, other apolipoproteins, lipolytic enzymes (such as LPL), and cell surface receptors [1–4]. It has long been known that, as with other small apolipoproteins, apoC-III binds to the surface of lipoproteins by virtue of being able to form amphipathic helices along the peptide chain, and recent results indicate that aromatic residues in the C-terminal half of apoC-III are especially important in mediating binding to TRLs [5]. The protein undergoes posttranslational modification resulting in three different isoforms containing zero, one, or two sialic acids (termed apoC-III_0_, apoC-III_1_ and apoC-III_2_) [6]. The physiological relevance of these glycoforms is still unclear, but the degree of sialylation of apoC-III has been proposed to influence lipoprotein lipase (LPL)-mediated hydrolysis of TRLs in the circulation. Recent investigations reveal that the different apoC-III glycoforms show specific patterns over the range of total apoC-III concentration, but the impact of this variation on the atherogenic potential of the apoprotein is unclear [7•].

ApoC-III is a key regulator of triglyceride metabolism, and human kinetic studies have shown that impaired catabolism of TRLs, linked to increased levels of plasma apoC-III, is the main determinant of plasma triglyceride levels in the population [8]. In accord with this concept, metabolic studies in hypertriglyceridemic subjects have demonstrated that apoB-containing lipoproteins are removed significantly less efficiently from the circulation if they are enriched in apoC-III [9]. It remains to be clarified if the impaired removal of TRLs and their remnants is mainly due to reduced lipolytic capacity or to reduced hepatic removal of TRL remnants. ApoC-III has also been proposed to directly influence plasma triglycerides by enhancing hepatic VLDL secretion [10–12]. Studies in genetically modified mice overexpressing apoC-III displayed increased hepatic secretion of VLDL particles [10]. However, inhibition of apoC-III synthesis using antisense oligonucleotides (ASO) in mice did not reduce VLDL secretion [13], so the biological significance of apoC-III as a regulator of hepatic VLDL assembly and release is still unclear, particularly in humans. A further illustration of this point comes from recent investigation of VLDL metabolism in subjects heterozygous for apoC-III deficiency. Here, individuals with half the level of apoC-III had the same VLDL apoB production rate as their unaffected siblings (who had normal apoC-III levels and higher plasma triglyceride) [14•].

**Fig. 1:**
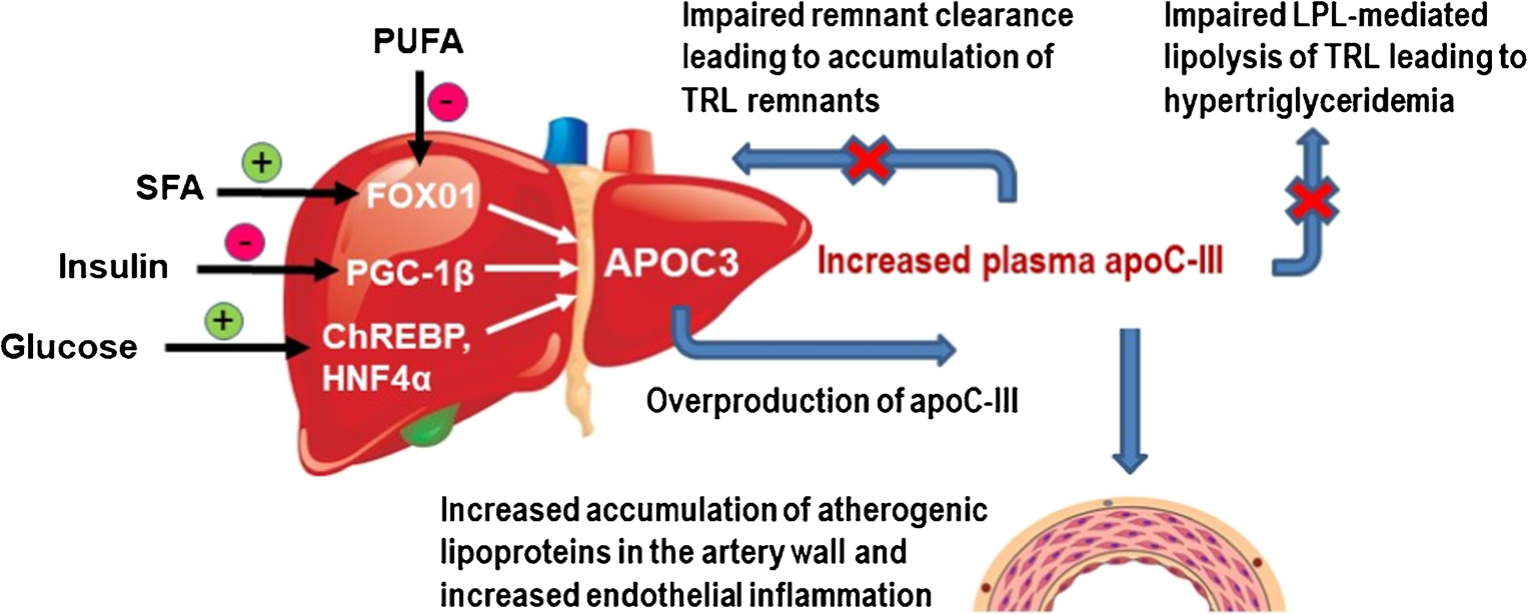
Proatherogenic action of apoC-III on lipid metabolism and atherogenicity. The APOC3 expression in hepatocytes is regulated by many metabolic and nutritional components, including circulating glucose, insulin, and fatty acids [15•]. Glucose induces increased expression of APOC3 via activation of the carbohydrate response element-binding protein (ChREBP) and hepatic nuclear factor-4a (HNF4a). Also, dietary intake of saturated fatty acid levels increased APOC3 expression by activation mainly of the peroxisome proliferator-activated receptor (PPAR) γ coactivator-1 β (PGC-1β). Insulin and dietary intake of polyunsaturated fatty acid (PUFA) represses by promoting phosphorylation of the nuclear transcription factor forkhead box O1 (FOXO1). Increased APOC3 expression induces increased plasma apoC-III concentration. ApoC-III induces increased plasma triglycerides and accumulation of triglyceride-rich lipoprotein (TRL) remnant particles by several mechanisms. These include inhibition of lipoprotein lipase (LPL)-mediated lipolysis of TRLs and preventing hepatic clearance of lipoprotein remnants. ApoC-III also exerts strong atherogenic functions through both indirect and direct mechanisms. These include increasing affinity of atherogenic lipoproteins to the extracellular matrix of the artery wall, leading to increased accumulation of atherogenic lipoproteins in the artery wall, and by promoting proinflammatory responses in endothelial cells and monocytes

There are several potential mechanisms whereby apoC-III can induce increased plasma triglycerides and accumulation of TRL remnant particles. These include inhibition of LPL-mediated lipolysis of TRLs, and impaired removal of TRL remnants (Fig. 1).

*Inhibition of LPL-mediated lipolysis of TRLs*—ApoC-III is a known inhibitor of lipoprotein lipase (LPL), the main enzyme responsible for the hydrolysis of triglyceride in TRLs. Underlying mechanisms include inhibiting TRL binding to the negatively charged cell surface where the enzyme is resident [16] and inhibiting activation of the enzyme by displacing the LPL activator apoC-II from the surface of the TRL particle [17]. This displacement is likely due to competition for available binding sites on the phospholipid monolayer surface of the lipoprotein; apoC-III is the most abundant apolipoprotein present at an average of 25–50 molecules per VLDL particle [18, 19].

*Preventing clearance of lipoprotein remnants*—Remnant particles, formed by partial lipolysis of TRLs, are rapidly removed from the circulation by the liver. The mechanism involves binding of remnants to heparan sulfate proteoglycans (HSPGs) and to members of the LDL receptor family on hepatocytes, followed by endocytosis. The ligand on the remnant particles is apolipoprotein E (apoE), and by displacing this protein from the lipoprotein particle surface (in analogy to the action on apoC-II above) [18], apoC-III can effectively impair the clearance of remnants [20]. Thus, the ratio of apoC-III (with its inhibitory role) and apoE (which mediates particle receptor-mediated clearance) on the remnant surface is potentially an important regulator of the metabolism of these lipoproteins [18].

In addition to effects on lipid metabolism, apoC-III has been shown to directly influence development of atherosclerosis by several routes including facilitating subendothelial accumulation of atherogenic lipoproteins by increasing their affinity for artery wall proteoglycans [21–26]. The mechanism of this interaction is complex since apoC-III itself does not bind artery wall proteoglycans, but seems to provoke changes in the lipid composition of the lipoproteins leading apoB to adopt a conformation that is more favorable for proteoglycan binding [23, 27]. ApoC-III may also promote aggregation and fusion of retained lipoproteins in the artery wall by activating sphingomyelinases (SMase) [28•, 29•]. In addition, apoC-III has been reported to facilitate interaction between monocytes and endothelial cells, promote smooth muscle cell proliferation, and induce inflammation by activating adhesion molecules and the proinflammatory nuclear factor-κB in monocytes and endothelial cells [30, 31].

### Lessons from Genetic Studies: Why Lowering of apoC-III Inhibition Should Be Pursued

Recent data from exome-wide association studies of lipids in > 300,000 individuals demonstrated a causal relationship between genetically determined elevated plasma triglyceride levels and coronary artery disease (CAD) [32•, 33•, 34]. The studies also provided evidence for the concept that increased lipolysis (i.e., LPL activity) may lead to reduced risk of atherosclerosis. Earlier success in demonstrating the impact on CAD risk of PCSK9 LOF mutations that led to LDL lowering paved the way for an examination of the consequences of LOF mutations of the apoC-III gene, and control of apoC-III as a prime lipid-lowering target [35]. The concept has also stimulated interest in other proteins that regulate lipolysis of TRLs including apoA-V and the Angptl family as alternate, novel therapeutic strategies.

Pollin et al. reported in 2008 that carriers of the APOC3 null mutation (R19X) had about 40% lower plasma apoC-III levels than non-carriers, significantly lower postprandial triglycerides, and higher HDL-cholesterol levels [36]—the R19X variant inserts a premature stop-codon in the mRNA transcript of the mutated gene [36]. As a non-invasive measure of prevalent atherosclerosis, the coronary artery calcification (CAC) score was determined in 1033 Amish subjects (representative of the background population in which the variant was found) and heterozygote carriers of the R19X null mutation. The latter had CAC scores significantly lower than non-carriers, and the authors therefore suggested that lifelong deficiency of apoC-III is cardioprotective [36]. This null variant was also reported to be associated with low plasma levels of apoC-III and triglycerides in a separate cohort on the island of Crete, with a frequency of about 2% [37•]. The allele frequency of the R19X variant is rare in the general population; 0.08% in Americans [38] and 0.05% in Europeans [39]. Recently, Reyes-Soffer reported that the 50% lower plasma apoC-III and 35% lower plasma triglyceride levels in R19X variant carriers were due to markedly higher clearance rates for VLDL particles indicating a faster lipolytic rate [14•]. As expected, the carriers of the R19X variant had lower apoC-III production rate but also increased apoC-III clearance rate. In this metabolic investigation, no influence of relative apoC-III deficiency was observed on direct VLDL clearance [14•].

The favorable lipid profile with low triglycerides and high HDL-cholesterol in subjects with APOC3 LOF mutations has been validated in a cohort (*n* = 80) recruited on the basis of high HDL-cholesterol levels (> 95th percentile) [40]; in 5 out of the 80 individuals, heterozygote LOF mutations in APOC3 (c.-13-2A > g, C.55 + 1G > A and Ala43Thr) were identified [40]. Thus, APOC3 mutations are enriched in individuals with hyperalphalipoproteinemia. The Ala43Thr variant has also been reported to associate with low apoC-III and plasma triglycerides in three individuals of Yucatan Indian descent [41]. Despite the findings quoted above from human turnover studies [14•], there are continued hints that enhanced lipolysis might not be the only mechanism for the lower plasma triglycerides in individuals with APOC3 LOF mutation, as Suddaram et al. reported that the Ala43Thr variant induces an impaired lipidation of nascent VLDL particles during the assembly of VLDL in the liver [42].

Two landmark studies published back-to-back in the New England Journal of Medicine in 2014 provided concrete evidence that apoC-III LOF variant carriers exhibit marked reductions in apoC-III, have lower plasma triglycerides, and experience significantly fewer CVD outcomes [43•, 44•]. These results were based on LOF mutations: the R19X nonsense mutation, two splice-site mutations (IVS2 + 1G → A and IVS3 + 1G → T), and the Ala43Thr missense mutation. The association of LOF carrier status with CVD outcomes was established independently in the NHLBI Exome Sequencing Project (*n* = 110,970) and in the Copenhagen Study (*n* = 75,725) [43•, 44•]. The fact that these large cohort studies show such similar results strengthens the findings considerably. Reduction in plasma triglycerides in both studies was about 40% in carriers compared to non-carriers, and this lipid difference was associated with marked reductions of CVD risk (approximately 40%) in both studies. These results suggest that 1 mg/dl decrease in apoC-III translates to a 4% decrease in CVD incidence [43•].

Recently, the power of LDL cholesterol and remnant cholesterol to predict ischemic vascular disease (IVD) risk was examined in apoC-III LOF carriers in a meta-analysis of 137,895 individuals [45]. The data demonstrated that the reduction of IVD risk in heterozygote carriers of apoC-III LOF mutations associated with reduction of remnant cholesterol (mean of − 43%) but not with change in LDL cholesterol (mean of − 4%) [45]. Apolipoprotein B, a marker of the total number of atherogenic lipoprotein particles in the circulation—each of VLDL, remnants, and LDL contains one apoB protein per particle—was 13% lower in LOF heterozygotes compared to non-carriers. In this context, it is noteworthy that in a recent evaluation of the association of the plasma triglyceride with CVD risk, Ference et al. [46•] reported, on the basis of large-scale Mendelian randomization analyses, that genetically determined change in triglyceride was not associated with altered CVD risk unless there was an accompanying perturbation in circulating apoB (i.e., particle) levels.

As these LOF mutations are rare, none of the early studies reported homozygosity of apoC-III LOF despite that fact that about 200,000 participants were examined. However, four individual homozygotes for the Arg19Thr mutation in apoC-III were recently identified in the PROMIS study which included 10,503 Pakistan participants [47•]. One of the LOF homozygote probands, his wife, and 27 first-degree relatives were invited for further investigations and genotyping. Unexpectedly, the wife also was found to be a LOF homozygote and, consequently, all nine children in the family were homozygotes for the Arg19Thr APOC3 variant [47•]. The plasma concentrations of apoC-III were extremely low in all homozygote family members as compared to heterozygotes and non-carriers. Fat feeding was used as a challenge to evaluate triglyceride metabolism in this condition; compared to non-carriers, homozygote family members showed a markedly blunted postprandial triglyceride elevation [47•]. These studies demonstrate critically that complete deficiency of apoC-III is not lethal, and that it is associated with very rapid lipolytic rates for TRLs.

### Can Dietary Modulation Reduce Plasma apoC-III Levels?

Glucose is known to modulate the hepatic expression of key enzymes of the glycolytic and lipogenic pathways [48], and Caron et al. have shown that glucose induces APOC3 expression in hepatocytes in vitro via a mechanism involving the transcription factors carbohydrate response element-binding protein and hepatocyte nuclear factor-4α [49] (Fig. 1). Interestingly, insulin and glucose seem to have opposite effects on apoC-III expression in human hepatocytes [49–51]. It has therefore been hypothesized that in insulin resistance associated with hyperglycemia, as in type 2 diabetes, insulin no longer represses APOC3 expression, whereas chronic glucose elevation enhances APOC3 expression, leading to increased plasma apoC-III levels and increased risk for atherosclerosis, via either induction of hypertriglyceridemia or other vascular effects of apoC-III [30, 52]. Thus, the influence of diets on cardiometabolic risk factors including apoC-III may differ depending on metabolic status. This and the fact that most dietary studies are small with different designs and dietary interventions may explain the inconsistent results presented [53]. That said, a large number of studies seem to indicate that diets enriched in carbohydrates (CHO) correlate with higher plasma apoC-III levels [54–58]. For example, high consumption of fructose induces adverse effects on cardiometabolic risk factors including increased plasma apoC-III levels [59, 60]. Fructose restriction, on the other hand, has been reported to reduce apoC-III concentrations [60, 61]. In line with these observations, we recently reported that a short-term intervention using an isocaloric low-carbohydrate diet induced a rapid, robust, and significant decline of apoC-III concentrations in addition to other metabolic benefits in obese subjects with NAFLD [62•].

Consumption of saturated fat seems to increase plasma apoC-III levels [63, 64] (Fig. 1). In contrast, dietary intake of mono- and unsaturated fat appears to reduce apoC-III concentrations [64]. Dietary intake of marine oils and omega-3 fatty acid preparations is linked to small, yet statistically significant improvements in lipoprotein profile of which the strongest effect is on plasma triglyceride concentration. Across studies, this effect was dose-dependent and related to studies’ mean baseline triglyceride [65]. Recent reports indicate that omega-3 PUFAs significantly decrease apoC-III [66•, 67•], and this may be a mechanism for their triglyceride-lowering effects [68]. In summary, available data are still inconsistent but dietary intervention may be used to reduce apoC-III concentrations; however, more research is needed.

### Available Therapies Reducing ApoC-III Plasma Concentrations

Both in vitro and animal studies indicate that fibrates as peroxisome proliferator-activated receptor alpha (PPARα) agonists reduce APOC3 expression resulting in lowering of plasma triglyceride [69–71]. In contrast, overexpression of human APOC3 in transgenic mice is associated with hypertriglyceridemia [72]. The underlying molecular mechanisms for how PPARα agonists influence APOC3 expression remain to be fully elucidated but seem to include a role for various co-factors that alter the PPAR-mediated transcriptional activation of target genes via complex signaling pathways [73]. The efficacy of different fibrates in lowering plasma triglyceride levels has been reported to be linked to their ability to reduce APOC3 expression in hepatocytes [74]. Overall, the response in APOC3 expression to fibrates is variable and depends on the agents’ efficacy and selectivity.

Surprisingly, no data exist on the responses of apoC-III levels in fibrate intervention trials [the Fenofibrate Intervention and Event Lowering in Diabetes (FIELD) trial; the Action to Control Cardiovascular Risk in Diabetes (ACCORD) trial; the Bezafibrate Infarction Prevention (BIP) trial; and the Veterans Affairs High-Density Lipoprotein Cholesterol Intervention Trial (VA-HIT)] addressing associations between CVD outcomes and changes in lipid profiles. Available data on changes of apoC-III levels are sparse and mainly from smaller studies with short duration and variable design, or from post hoc analyses. The reduction of apoC-III on fibrates in clinical studies seems to average about 20%, but there is a wide range from 10 to 40% [75–79]. Data on how PPARγ agonists (pioglitazone, rosiglitazone) affect apoC-III metabolism in humans are even scantier and partially conflicting as pioglitazone seems to reduce plasma apoC-III levels whereas rosiglitazone appears to have the opposite effect [80, 81].

Interestingly, there is robust evidence to indicate that both nicotinic acid (niacin) and statin therapy can reduce hepatic APOC3 expression. Hernandez et al. reported that the peroxisome proliferator-activated receptor gamma coactivator 1-beta (PGC-1β), a transcriptional cofactor, regulates the expression of APOC3 [82]; nicotinic acid has been shown to reduce PGC-1β expression and this leads in turn to a decrease in APOC3 expression. In human kinetic studies, Ooi et al. reported that rosuvastatin not only decreased the production rate of apoC-III but also increased its catabolism resulting in a reduced VLDL-apoC-III concentration [83]. In accord with these mechanistic linkages, plasma apoC-III levels were found to be significantly reduced by statins in a meta-analysis of well-controlled clinical trials [84].

Omega-3 carboxylic acids (OM3-CA) have been shown to reduce plasma apoC-III relative to placebo, as well as apoC-III in HDL, and apoC-III in LDL [67•, 85, 86]. Unexpectedly, OM3-CA selectively also increase the concentration of LDL that does not contain apoC-III, a subspecies with, reportedly, a weak relation to coronary heart disease [67•]. In line with these findings, a recent meta-analysis (*n* = 2062) reported that omega-3 PUFA in doses > 2 g for 7 days was associated with significant reductions of apoC-III levels [66, 87]. The reduction of apoC-III varied between 20 and 30%. However, it remains to be proven if the apoC-III reduction is replicated in the recently published REDUCE-IT and VITAL trials, or indeed is found to contribute to clinical benefit [88, 89].

### Pipeline Options Targeting for ApoC-III Inhibition

As apoC-III has emerged as an attractive target, several novel technologies have been devised to regulate its concentration in cells and in the circulation. These include small antisense oligonucleotides (ASOs), interfering RNAs (siRNAs), and monoclonal antibodies [90, 91]. RNA interference and related RNA silencing techniques provide unprecedented opportunities to control gene expression in vivo [92], as they selectively silence translation of their target messenger RNAs [93, 94].

The translation of these silencing technologies from basic science laboratories to the clinic has been speedy. Several discoveries have underpinned this phenomenon; of particular importance is the development of novel targeting strategies. For example, binding GalNAc moieties—the ligand of the hepatic asialoglycoprotein receptor—to siRNA or ASO [95, 96] enables efficient RNAi-mediated gene silencing to occur specifically in hepatocytes and enhances the potency of the agents about 30-fold [97]. Again, PCSK9 inhibition has been a pathfinder in this new lipid-lowering therapeutic approach. Inclisiran, a subcutaneously administered investigational RNAi therapeutic (ALN-PCSsc), targeting PCSK9 for the treatment of hypercholesterolemia successfully lowers LDL over the long term and is presently in phase III development. Volanesorsen (IONIS-APOCIII Rx) represents a second-generation antisense nucleotide designed to specifically bind to apoC-III mRNA. Volanesorsen was reported to reduce effectively APOC3 expression and plasma triglycerides in rodent models and nonhuman primates [13]. The phase I clinical study was a double-blind placebo-controlled, dose-ranging study in 25 healthy subjects. It showed a dose-dependent reduction of apoC-III of up to 90% and concomitant marked reduction in plasma triglycerides. Likewise, volanesorsen resulted in marked reduction of plasma triglycerides by 56 to 86% in three patients with familial chylomicron syndrome (FCS) with triglycerides ranging from 15.9 to 23.5 mmol/l due to genetic defects in LPL [98•]. The reduction of apoC-III varied from 71 to 90% after 13 weeks of the therapy, and, importantly from a clinical efficacy point of view, all three patients had plasma triglycerides less than 5.7 mmol/l after the therapy thereby diminishing considerably the risk of pancreatitis. These dramatic results strongly indicate that apoC-III not only inhibits LPL activity but has a suppressive action on the overall removal of TRLs by LPL-independent pathways. Further evidence was provided in murine studies using an apoC-III ASO showing that apoC-III inhibits the hepatic clearance of TRLs by the LDLR/LRP1 pathways [99•]. The seminal role of these LPL-independent pathways was confirmed by further studies in subjects with LPL deficiency [100].

These proof-of-concept studies were followed by a rapid initiation of phase II dose-ranging trials using volanesorsen as a monotherapy or as an add-on to stable fibrate therapy for 13 weeks [101•]. This cohort (*n* = 57) included untreated patients with a wide range of plasma triglycerides (from 4.0 to 22.6 mmol/l). The results showed that decreases of both plasma apoC-III and triglycerides were dose-dependent, averaging about 80% and 71% respectively. The simultaneous increase of HDL-cholesterol was about 46%. Volanesorsen was also tested in a randomized, double-blind, placebo-controlled trial in 15 overweight or obese subjects with type 2 diabetes [102]. Patients had HbA1c > 7.5% on a stable dose of metformin (> 1000 mg/day) and plasma triglycerides between 200 and 500 mg/dl and were randomized in a 2:1 ratio to receive volanesorsen 300 mg or matched placebo for 15 weekly doses. Volanesorsen markedly improved dyslipidaemia by reducing both apoC-III (− 88%) and plasma TG (− 69%) and increased HDL-cholesterol by 42% after 13 weeks. Notably, the plasma triglyceride level averaged 2.8 mmol/l (249 mg/dl) at baseline, and all patients achieved levels below 0.84 mmol/l (< 76 mg/dl) at the end of the treatment period. A two-step hyperinsulinemic-euglycemic clamp was performed before and after the treatment period, and it was found that volanesorsen improved whole-body insulin sensitivity by about 57% as compared to placebo. The investigators reported a significant relationship between improved insulin sensitivity and plasma apoC-III suppression. Notably, HbA1c significantly improved during the volanesorsen treatment. These data suggest that effective suppression of plasma triglycerides by inhibition of apoC-III might be a promising strategy in management of diabetic dyslipidaemia.

Two randomized, double-blind, placebo-controlled phase 3 trials with volanesorsen have been performed. In the APPROACH trial, 66 patients with documented FCS and with fasting triglycerides > 8.5 mmol/L (750 mg/dl) on restricted low-fat diet were randomized to receive either active drug 300 mg once a week or matched placebo for 52 weeks [103]. In the COMPASS study which recruited 113 subjects with severe hypertriglyceridemia between 5.7 and 14.8 mmol/l (500–1261 mg/dl) [90], patients were randomized in a 2:1 ratio to receive either volanesorsen or placebo once weekly for 26 weeks. In both studies, the efficacy of volanesorsen to reduce both apoC-III and triglyceride concentrations was remarkable, averaging about 70–80%. As severe hypertriglyceridemia associates with high risk of acute pancreatitis, the data from the two studies were combined to clarify if volanesorsen therapy can lower the risk of this serious condition [104]. The episodes of acute pancreatitis were markedly less in patients treated with active drug than in the placebo group.

Despite the fact that FCS is a severe disorder and there are no approved drugs to prevent the attendant high risk of pancreatitis, the FDA did not approve volanesorsen for the treatment of FCS as a rare disease (August 2018) [105]. However, in March 2019, the Committee for Medicinal Products for Human Use (CHMP) of the European Medicines Agency (EMA) adopted a positive opinion recommending conditional marketing authorization for Waylivra (volanesorsen) as an adjunct to diet in adult patients with genetically confirmed FCS who are at high risk for pancreatitis, in whom response to diet and triglyceride-lowering therapy has been inadequate.

To complete this therapeutic overview, it should be noted that apoC-III (again like PCSK9) can also be targeted by monoclonal antibodies, and it has been proposed that anti-apoC-III monoclonal antibodies would prevent binding of apoC-III to lipoproteins resulting in enhanced clearance of lipoprotein-free apoC-III via the kidney [106•].

### Safety Concerns of ApoC-III Inhibition by Antisense Oligonucleotide Therapy

The negative decision by FDA to approve volanesorsen for the treatment of FCS as a rare disease was based on safety concerns. Data from phase II–III clinical studies indicated that volanesorsen therapy was well tolerated overall with major side effects being local reactions at injection sites; the incidence of these varied between 10 and 23%, and they were in general mild to moderate and resolved rapidly. More important was the occurrence of thrombocytopenia and resulting potential for serious bleeding that was reported in the APPROACH study. Grade 4 thrombocytopenia occurred in three patients, which ended after they stopped taking the drug. None of these patients had any major bleeding events, and all recovered to normal platelet count following drug discontinuation and administration of corticosteroids. Furthermore, there were no withdrawals due to platelet counts after the company began monitoring the side effect. In contrast, no significant reduction of platelet counts was reported in the COMPASS study [15•, 95], and current thinking is that underlying mechanism seems to be linked to the drug delivery methodology and not to apoC-III per se, as another 2′-MOE modified antisense oligonucleotide has been reported to cause dose-dependent reduction of platelets in monkeys and humans [107]. These potential issues may be addressed by the conjugation of GalNAc to ASO targeting apoC-III which as mentioned above enables liver-specific delivery of the anti-apoC-III ASO and enhanced potency allowing lower doses to be used [95, 108]. Pilot data in healthy volunteers have shown that IONIS-APOCIII LRx, a GalNAc3 conjugated APOC3 antisense oligonucleotide, has comparable potency to volanesorsen to lower both plasma apoC-III and TG levels, but without significant side effect or platelet reduction [109]. Results seem promising, but whether the safety and tolerability will be improved in larger clinical studies remains to be demonstrated.

## Conclusions

Overall, the available data suggest that the lowering of apoC-III and associated change in plasma triglycerides and remnant cholesterol can be protective against progression of atherosclerosis reflected in reduced CVD risk. Consequently, the inhibition of apoC-III synthesis and reduction of plasma apoC-III levels has become an attractive therapeutic target to reduce the residual risk in statin-treated subjects. The future will evidence if the translation of current “bench” knowledge on apoC-III pathophysiology to “bedside” will be as rapid as for the development of PCSK9 inhibitors; will apoC-III be the next PCSK9?

## References

[1] Gangabadage CS, Zdunek J, Tessari M, Nilsson S, Olivecrona G, Wijmenga SS (2008). Structure and dynamics of human apolipoprotein CIII. J Biol Chem.

[2] Liu H, Talmud PJ, Lins L, Brasseur R, Olivecrona G, Peelman F, Vandekerckhove J, Rosseneu M, Labeur C (2000). Characterization of recombinant wild type and site-directed mutations of apolipoprotein C-III: lipid binding, displacement of ApoE, and inhibition of lipoprotein lipase. Biochemistry..

[3] Sparrow JT, Pownall HJ, Hsu FJ, Blumenthal LD, Culwell AR, Gotto AM (1977). Lipid binding by fragments of apolipoprotein C-III-1 obtained by thrombin cleavage. Biochemistry..

[4] Lins L, Flore C, Chapelle L, Talmud PJ, Thomas A, Brasseur R (2002). Lipid-interacting properties of the N-terminal domain of human apolipoprotein C-III. Protein Eng.

[5] Meyers NL, Larsson M, Vorrsjo E, Olivecrona G, Small DM (2017). Aromatic residues in the C terminus of apolipoprotein C-III mediate lipid binding and LPL inhibition. J Lipid Res.

[6] Trenchevska O, Schaab MR, Nelson RW, Nedelkov D (2015). Development of multiplex mass spectrometric immunoassay for detection and quantification of apolipoproteins C-I, C-II, C-III and their proteoforms. Methods..

[7] Olivieri O, Chiariello C, Martinelli N, Castagna A, Speziali G, Girelli D (2018). Sialylated isoforms of apolipoprotein C-III and plasma lipids in subjects with coronary artery disease. Clin Chem Lab Med.

[8] Boren J, Watts GF, Adiels M, Soderlund S, Chan DC, Hakkarainen A (2015). Kinetic and related determinants of plasma triglyceride concentration in abdominal obesity: multicenter tracer kinetic study. Arterioscler Thromb Vasc Biol.

[9] Zheng C, Khoo C, Furtado J, Sacks FM (2010). Apolipoprotein C-III and the metabolic basis for hypertriglyceridemia and the dense low-density lipoprotein phenotype. Circulation..

[10] Yao Z (2012). Human apolipoprotein C-III - a new intrahepatic protein factor promoting assembly and secretion of very low density lipoproteins. Cardiovasc Hematol Disord Drug Targets.

[11] Taskinen MR, Boren J (2015). New insights into the pathophysiology of dyslipidemia in type 2 diabetes. Atherosclerosis..

[12] Adiels M, Olofsson SO, Taskinen MR, Boren J (2008). Overproduction of very low-density lipoproteins is the hallmark of the dyslipidemia in the metabolic syndrome. Arterioscler Thromb Vasc Biol.

[13] Graham MJ, Lee RG, Bell TA, Fu W, Mullick AE, Alexander VJ (2013). Antisense oligonucleotide inhibition of apolipoprotein C-III reduces plasma triglycerides in rodents, nonhuman primates, and humans. Circ Res.

[14] Reyes-Soffer G, Sztalryd C, Horenstein RB, Holleran S, Matveyenko A, Thomas T (2019). Effects of APOC3 heterozygous deficiency on plasma lipid and lipoprotein metabolism. Arterioscler Thromb Vasc Biol.

[15] Ramms B, Gordts P (2018). Apolipoprotein C-III in triglyceride-rich lipoprotein metabolism. Curr Opin Lipidol.

[16] Ebara T, Ramakrishnan R, Steiner G, Shachter NS (1997). Chylomicronemia due to apolipoprotein CIII overexpression in apolipoprotein E-null mice. Apolipoprotein CIII-induced hypertriglyceridemia is not mediated by effects on apolipoprotein E. J Clin Invest.

[17] Lambert DA, Smith LC, Pownall H, Sparrow JT, Nicolas JP, Gotto AM (2000). Hydrolysis of phospholipids by purified milk lipoprotein lipase. Effect of apoprotein CII, CIII, A and E, and synthetic fragments. Clin Chim Acta.

[18] Sacks FM (2015). The crucial roles of apolipoproteins E and C-III in apoB lipoprotein metabolism in normolipidemia and hypertriglyceridemia. Curr Opin Lipidol.

[19] Campos H, Perlov D, Khoo C, Sacks FM (2001). Distinct patterns of lipoproteins with apoB defined by presence of apoE or apoC-III in hypercholesterolemia and hypertriglyceridemia. J Lipid Res.

[20] Narayanaswami V, Ryan RO (2000). Molecular basis of exchangeable apolipoprotein function. Biochim Biophys Acta.

[21] Olin-Lewis K, Krauss RM, La Belle M, Blanche PJ, Barrett PH, Wight TN (2002). ApoC-III content of apoB-containing lipoproteins is associated with binding to the vascular proteoglycan biglycan. J Lipid Res.

[22] Davidsson P, Hulthe J, Fagerberg B, Olsson BM, Hallberg C, Dahllof B (2005). A proteomic study of the apolipoproteins in LDL subclasses in patients with the metabolic syndrome and type 2 diabetes. J Lipid Res.

[23] Hiukka A, Stahlman M, Pettersson C, Levin M, Adiels M, Teneberg S, Leinonen ES, Hulten LM, Wiklund O, Oresic M, Olofsson SO, Taskinen MR, Ekroos K, Boren J (2009). ApoCIII-enriched LDL in type 2 diabetes displays altered lipid composition, increased susceptibility for sphingomyelinase, and increased binding to biglycan. Diabetes..

[24] Boren J, Gustafsson M, Skalen K, Flood C, Innerarity TL (2000). Role of extracellular retention of low density lipoproteins in atherosclerosis. Curr Opin Lipidol.

[25] Gustafsson M, Boren J (2004). Mechanism of lipoprotein retention by the extracellular matrix. Curr Opin Lipidol.

[26] Gustafsson M, Flood C, Jirholt P, Boren J (2004). Retention of atherogenic lipoproteins in atherogenesis. Cell Mol Life Sci.

[27] Segrest J, Jones M, Mishra V, Pierotti V, Young S, Boren J (1998). Apolipoprotein B-100: conservation of lipid-associating amphipathic secondary structural motifs in nine species of vertebrates. J Lipid Res.

[28] Schissel SL, Jiang X, Tweedie-Hardman J, Jeong T, Camejo EH, Najib J, Rapp JH, Williams KJ, Tabas I (1998). Secretory sphingomyelinase, a product of the acid sphingomyelinase gene, can hydrolyze atherogenic lipoproteins at neutral pH. Implications for atherosclerotic lesion development. J Biol Chem.

[29] Ruuth M, Nguyen SD, Vihervaara T, Hilvo M, Laajala TD, Kondadi PK (2018). Susceptibility of low-density lipoprotein particles to aggregate depends on particle lipidome, is modifiable, and associates with future cardiovascular deaths. Eur Heart J.

[30] Kawakami A, Aikawa M, Alcaide P, Luscinskas FW, Libby P, Sacks FM (2006). Apolipoprotein CIII induces expression of vascular cell adhesion molecule-1 in vascular endothelial cells and increases adhesion of monocytic cells. Circulation..

[31] Kawakami A, Aikawa M, Nitta N, Yoshida M, Libby P, Sacks FM (2007). Apolipoprotein CIII-induced THP-1 cell adhesion to endothelial cells involves pertussis toxin-sensitive G protein- and protein kinase C alpha-mediated nuclear factor-kappaB activation. Arterioscler Thromb Vasc Biol.

[32] Liu DJ, Peloso GM, Yu H, Butterworth AS, Wang X, Mahajan A (2017). Exome-wide association study of plasma lipids in > 300,000 individuals. Nat Genet.

[33] Klarin D, Damrauer SM, Cho K, Sun YV, Teslovich TM, Honerlaw J (2018). Genetics of blood lipids among ~300,000 multi-ethnic participants of the Million Veteran Program. Nat Genet.

[34] Tall AR (2017). Increasing lipolysis and reducing atherosclerosis. N Engl J Med.

[35] Bernelot Moens SJ, van Capelleveen JC, Stroes ES (2014). Inhibition of ApoCIII: the next PCSK9?. Curr Opin Lipidol.

[36] Pollin TI, Damcott CM, Shen H, Ott SH, Shelton J, Horenstein RB, Post W, McLenithan JC, Bielak LF, Peyser PA, Mitchell BD, Miller M, O'Connell JR, Shuldiner AR (2008). A null mutation in human APOC3 confers a favorable plasma lipid profile and apparent cardioprotection. Science..

[37] Tachmazidou I, Dedoussis G, Southam L, Farmaki AE, Ritchie GR, Xifara DK (2013). A rare functional cardioprotective APOC3 variant has risen in frequency in distinct population isolates. Nat Commun.

[38] Crawford DC, Dumitrescu L, Goodloe R, Brown-Gentry K, Boston J, McClellan B, Sutcliffe C, Wiseman R, Baker P, Pericak-Vance MA, Scott WK, Allen M, Mayo P, Schnetz-Boutaud N, Dilks HH, Haines JL, Pollin TI (2014). Rare variant APOC3 R19X is associated with cardio-protective profiles in a diverse population-based survey as part of the epidemiologic architecture for genes linked to environment study. Circ Cardiovasc Genet.

[39] Muddyman D, Smee C, Griffin H, Kaye J (2013). Implementing a successful data-management framework: the UK10K managed access model. Genome Med.

[40] Bochem AE, van Capelleveen JC, Dallinga-Thie GM, Schimmel AW, Motazacker MM, Tietjen I (2014). Two novel mutations in apolipoprotein C3 underlie atheroprotective lipid profiles in families. Clin Genet.

[41] Liu H, Labeur C, Xu CF, Ferrell R, Lins L, Brasseur R, Rosseneu M, Weiss KM, Humphries SE, Talmud PJ (2000). Characterization of the lipid-binding properties and lipoprotein lipase inhibition of a novel apolipoprotein C-III variant Ala23Thr. J Lipid Res.

[42] Sundaram M, Curtis KR, Amir Alipour M, LeBlond ND, Margison KD, Yaworski RA (2017). The apolipoprotein C-III (Gln38Lys) variant associated with human hypertriglyceridemia is a gain-of-function mutation. J Lipid Res.

[43] Blood I, Crosby J, Peloso GM, Auer PL, Tg, Hdl Working Group of the Exome Sequencing Project NHL (2014). Loss-of-function mutations in APOC3, triglycerides, and coronary disease. N Engl J Med.

[44] Jorgensen AB, Frikke-Schmidt R, Nordestgaard BG, Tybjaerg-Hansen A (2014). Loss-of-function mutations in APOC3 and risk of ischemic vascular disease. N Engl J Med.

[45] Wulff AB, Nordestgaard BG, Tybjaerg-Hansen A (2018). APOC3 loss-of-function mutations, remnant cholesterol, low-density lipoprotein cholesterol, and cardiovascular risk: mediation- and meta-analyses of 137 895 individuals. Arterioscler Thromb Vasc Biol.

[46] Ference BA, Kastelein JJP, Ray KK, Ginsberg HN, Chapman MJ, Packard CJ (2019). Association of triglyceride-lowering LPL variants and LDL-C-lowering LDLR variants with risk of coronary heart disease. JAMA.

[47] Saleheen D, Natarajan P, Armean IM, Zhao W, Rasheed A, Khetarpal SA (2017). Human knockouts and phenotypic analysis in a cohort with a high rate of consanguinity. Nature.

[48] Shih HM, Liu Z, Towle HC (1995). Two CACGTG motifs with proper spacing dictate the carbohydrate regulation of hepatic gene transcription. J Biol Chem.

[49] Caron S, Verrijken A, Mertens I, Samanez CH, Mautino G, Haas JT, Duran-Sandoval D, Prawitt J, Francque S, Vallez E, Muhr-Tailleux A, Berard I, Kuipers F, Kuivenhoven JA, Biddinger SB, Taskinen MR, van Gaal L, Staels B (2011). Transcriptional activation of apolipoprotein CIII expression by glucose may contribute to diabetic dyslipidemia. Arterioscler Thromb Vasc Biol.

[50] Altomonte J, Cong L, Harbaran S, Richter A, Xu J, Meseck M, Dong HH (2004). Foxo1 mediates insulin action on apoC-III and triglyceride metabolism. J Clin Invest.

[51] Chen M, Breslow JL, Li W, Leff T (1994). Transcriptional regulation of the apoC-III gene by insulin in diabetic mice: correlation with changes in plasma triglyceride levels. J Lipid Res.

[52] Taskinen MR, Boren J (2016). Why is apolipoprotein CIII emerging as a novel therapeutic target to reduce the burden of cardiovascular disease?. Curr Atheroscler Rep.

[53] Choo VL, Viguiliouk E, Blanco Mejia S, Cozma AI, Khan TA, Ha V (2018). Food sources of fructose-containing sugars and glycaemic control: systematic review and meta-analysis of controlled intervention studies. BMJ..

[54] Huff MW, Nestel PJ (1982). Metabolism of apolipoproteins CII, CIII1, CIII2 and VLDL-B in human subjects consuming high carbohydrate diets. Metabolism..

[55] Archer WR, Desroches S, Lamarche B, Deriaz O, Landry N, Fontaine-Bisson B (2005). Variations in plasma apolipoprotein C-III levels are strong correlates of the triglyceride response to a high-monounsaturated fatty acid diet and a high-carbohydrate diet. Metabolism..

[56] Furtado JD, Campos H, Appel LJ, Miller ER, Laranjo N, Carey VJ, Sacks FM (2008). Effect of protein, unsaturated fat, and carbohydrate intakes on plasma apolipoprotein B and VLDL and LDL containing apolipoprotein C-III: results from the OmniHeart trial. Am J Clin Nutr.

[57] Shin MJ, Blanche PJ, Rawlings RS, Fernstrom HS, Krauss RM (2007). Increased plasma concentrations of lipoprotein(a) during a low-fat, high-carbohydrate diet are associated with increased plasma concentrations of apolipoprotein C-III bound to apolipoprotein B-containing lipoproteins. Am J Clin Nutr.

[58] Mendoza S, Trenchevska O, King SM, Nelson RW, Nedelkov D, Krauss RM, Yassine HN (2017). Changes in low-density lipoprotein size phenotypes associate with changes in apolipoprotein C-III glycoforms after dietary interventions. J Clin Lipidol.

[59] Taskinen MR, Soderlund S, Bogl LH, Hakkarainen A, Matikainen N, Pietilainen KH (2017). Adverse effects of fructose on cardiometabolic risk factors and hepatic lipid metabolism in subjects with abdominal obesity. J Intern Med.

[60] Stanhope KL, Medici V, Bremer AA, Lee V, Lam HD, Nunez MV, Chen GX, Keim NL, Havel PJ (2015). A dose-response study of consuming high-fructose corn syrup-sweetened beverages on lipid/lipoprotein risk factors for cardiovascular disease in young adults. Am J Clin Nutr.

[61] Schwarz JM, Noworolski SM, Erkin-Cakmak A, Korn NJ, Wen MJ, Tai VW, Jones GM, Palii SP, Velasco-Alin M, Pan K, Patterson BW, Gugliucci A, Lustig RH, Mulligan K (2017). Effects of dietary fructose restriction on liver fat, De novo lipogenesis, and insulin kinetics in children with obesity. Gastroenterology..

[62] Mardinoglu A, Wu H, Bjornson E, Zhang C, Hakkarainen A, Rasanen SM (2018). An integrated understanding of the rapid metabolic benefits of a carbohydrate-restricted diet on hepatic steatosis in humans. Cell Metab.

[63] Pavlic M, Valero R, Duez H, Xiao C, Szeto L, Patterson BW (2008). Triglyceride-rich lipoprotein-associated apolipoprotein C-III production is stimulated by plasma free fatty acids in humans. Arterioscler Thromb Vasc Biol.

[64] Faghihnia N, Mangravite LM, Chiu S, Bergeron N, Krauss RM (2012). Effects of dietary saturated fat on LDL subclasses and apolipoprotein CIII in men. Eur J Clin Nutr.

[65] Balk Ethan, Lichtenstein Alice (2017). Omega-3 Fatty Acids and Cardiovascular Disease: Summary of the 2016 Agency of Healthcare Research and Quality Evidence Review. Nutrients.

[66] Sahebkar A, Simental-Mendia LE, Mikhailidis DP, Pirro M, Banach M, Sirtori CR (2018). Effect of omega-3 supplements on plasma apolipoprotein C-III concentrations: a systematic review and meta-analysis of randomized controlled trials. Ann Med.

[67] Morton AM, Furtado JD, Lee J, Amerine W, Davidson MH, Sacks FM (2016). The effect of omega-3 carboxylic acids on apolipoprotein CIII-containing lipoproteins in severe hypertriglyceridemia. J Clin Lipidol.

[68] Oscarsson J, Hurt-Camejo E (2017). Omega-3 fatty acids eicosapentaenoic acid and docosahexaenoic acid and their mechanisms of action on apolipoprotein B-containing lipoproteins in humans: a review. Lipids Health Dis.

[69] Hertz R, Bishara-Shieban J, Bar-Tana J (1995). Mode of action of peroxisome proliferators as hypolipidemic drugs. Suppression of apolipoprotein C-III. J Biol Chem.

[70] Ito Y, Azrolan N, O'Connell A, Walsh A, Breslow JL (1990). Hypertriglyceridemia as a result of human apo CIII gene expression in transgenic mice. Science..

[71] Staels B, Vu-Dac N, Kosykh VA, Saladin R, Fruchart JC, Dallongeville J, Auwerx J (1995). Fibrates downregulate apolipoprotein C-III expression independent of induction of peroxisomal acyl coenzyme A oxidase. A potential mechanism for the hypolipidemic action of fibrates. J Clin Invest.

[72] Aalto-Setala K, Fisher EA, Chen X, Chajek-Shaul T, Hayek T, Zechner R (1992). Mechanism of hypertriglyceridemia in human apolipoprotein (apo) CIII transgenic mice. Diminished very low density lipoprotein fractional catabolic rate associated with increased apo CIII and reduced apo E on the particles. J Clin Invest.

[73] Bougarne N, Weyers B, Desmet SJ, Deckers J, Ray DW, Staels B, de Bosscher K (2018). Molecular actions of PPARalpha in lipid metabolism and inflammation. Endocr Rev.

[74] Haubenwallner S, Essenburg AD, Barnett BC, Pape ME, DeMattos RB, Krause BR, Minton LL, Auerbach BJ, Newton RS, Leff T (1995). Hypolipidemic activity of select fibrates correlates to changes in hepatic apolipoprotein C-III expression: a potential physiologic basis for their mode of action. J Lipid Res.

[75] Reyes-Soffer G, Ngai CI, Lovato L, Karmally W, Ramakrishnan R, Holleran S, Ginsberg HN (2013). Effect of combination therapy with fenofibrate and simvastatin on postprandial lipemia in the ACCORD lipid trial. Diabetes Care.

[76] Attia N, Durlach V, Cambilleau M, Roche D, Girard-Globa A (2000). Postprandial concentrations and distribution of apo C-III in type 2 diabetic patients. Effect of bezafibrate treatment. Atherosclerosis..

[77] Lemieux I, Salomon H, Despres JP (2003). Contribution of apo CIII reduction to the greater effect of 12-week micronized fenofibrate than atorvastatin therapy on triglyceride levels and LDL size in dyslipidemic patients. Ann Med.

[78] de Man FH, de Beer F, van der Laarse A, Jansen H, Leuven JA, Souverijn JH (2000). The hypolipidemic action of bezafibrate therapy in hypertriglyceridemia is mediated by upregulation of lipoprotein lipase: no effects on VLDL substrate affinity to lipolysis or LDL receptor binding. Atherosclerosis..

[79] Chan DC, Watts GF, Ooi EM, Ji J, Johnson AG, Barrett PH (2008). Atorvastatin and fenofibrate have comparable effects on VLDL-apolipoprotein C-III kinetics in men with the metabolic syndrome. Arterioscler Thromb Vasc Biol.

[80] Nagashima K, Lopez C, Donovan D, Ngai C, Fontanez N, Bensadoun A, Fruchart-Najib J, Holleran S, Cohn JS, Ramakrishnan R, Ginsberg HN (2005). Effects of the PPARgamma agonist pioglitazone on lipoprotein metabolism in patients with type 2 diabetes mellitus. J Clin Invest.

[81] Wagner JA, Larson PJ, Weiss S, Miller JL, Doebber TW, Wu MS, Moller DE, Gottesdiener KM (2005). Individual and combined effects of peroxisome proliferator-activated receptor and {gamma} agonists, fenofibrate and rosiglitazone, on biomarkers of lipid and glucose metabolism in healthy nondiabetic volunteers. J Clin Pharmacol.

[82] Hernandez C, Molusky M, Li Y, Li S, Lin JD (2010). Regulation of hepatic ApoC3 expression by PGC-1beta mediates hypolipidemic effect of nicotinic acid. Cell Metab.

[83] Ooi EM, Watts GF, Chan DC, Chen MM, Nestel PJ, Sviridov D (2008). Dose-dependent effect of rosuvastatin on VLDL-apolipoprotein C-III kinetics in the metabolic syndrome. Diabetes Care.

[84] Sahebkar A, Simental-Mendia LE, Mikhailidis DP, Pirro M, Banach M, Sirtori CR (2018). Effect of statin therapy on plasma apolipoprotein CIII concentrations: a systematic review and meta-analysis of randomized controlled trials. J Clin Lipidol.

[85] Maki KC, Bays HE, Dicklin MR, Johnson SL, Shabbout M (2011). Effects of prescription omega-3-acid ethyl esters, coadministered with atorvastatin, on circulating levels of lipoprotein particles, apolipoprotein CIII, and lipoprotein-associated phospholipase A2 mass in men and women with mixed dyslipidemia. J Clin Lipidol.

[86] Dunbar RL, Nicholls SJ, Maki KC, Roth EM, Orloff DG, Curcio D, Johnson J, Kling D, Davidson MH (2015). Effects of omega-3 carboxylic acids on lipoprotein particles and other cardiovascular risk markers in high-risk statin-treated patients with residual hypertriglyceridemia: a randomized, controlled, double-blind trial. Lipids Health Dis.

[87] Ballantyne CM, Bays HE, Braeckman RA, Philip S, Stirtan WG, Doyle RT, Soni PN, Juliano RA (2016). Icosapent ethyl (eicosapentaenoic acid ethyl ester): effects on plasma apolipoprotein C-III levels in patients from the MARINE and ANCHOR studies. J Clin Lipidol.

[88] Bhatt DL, Steg PG, Miller M, Brinton EA, Jacobson TA, Ketchum SB, Doyle RT Jr, Juliano RA, Jiao L, Granowitz C, Tardif JC, Ballantyne CM, REDUCE-IT Investigators (2019). Cardiovascular risk reduction with icosapent ethyl for hypertriglyceridemia. N Engl J Med.

[89] Bowman L, Mafham M, Wallendszus K, Stevens W, Buck G, Group ASC (2018). Effects of n-3 fatty acid supplements in diabetes mellitus. N Engl J Med.

[90] Gouni-Berthold I (2017). The role of antisense oligonucleotide therapy against apolipoprotein-CIII in hypertriglyceridemia. Atheroscler Suppl.

[91] Watts JK, Corey DR (2012). Silencing disease genes in the laboratory and the clinic. J Pathol.

[92] Mello CC, Conte D (2004). Revealing the world of RNA interference. Nature..

[93] Carthew RW, Sontheimer EJ (2009). Origins and mechanisms of miRNAs and siRNAs. Cell..

[94] Bernards R (2006). Exploring the uses of RNAi--gene knockdown and the Nobel prize. N Engl J Med.

[95] Crooke ST, Witztum JL, Bennett CF, Baker BF (2018). RNA-targeted therapeutics. Cell Metab.

[96] Springer AD, Dowdy SF (2018). GalNAc-siRNA conjugates: leading the way for delivery of RNAi therapeutics. Nucleic Acid Ther.

[97] Schmidt K, Prakash TP, Donner AJ, Kinberger GA, Gaus HJ, Low A, Østergaard ME, Bell M, Swayze EE, Seth PP (2017). Characterizing the effect of GalNAc and phosphorothioate backbone on binding of antisense oligonucleotides to the asialoglycoprotein receptor. Nucleic Acids Res.

[98] Gaudet D, Brisson D, Tremblay K, Alexander VJ, Singleton W, Hughes SG (2014). Targeting APOC3 in the familial chylomicronemia syndrome. N Engl J Med.

[99] Gordts PL, Nock R, Son NH, Ramms B, Lew I, Gonzales JC (2016). ApoC-III inhibits clearance of triglyceride-rich lipoproteins through LDL family receptors. J Clin Invest.

[100] Yang X, Lee SR, Choi YS, Alexander VJ, Digenio A, Yang Q, Miller YI, Witztum JL, Tsimikas S (2016). Reduction in lipoprotein-associated apoC-III levels following volanesorsen therapy: phase 2 randomized trial results. J Lipid Res.

[101] Gaudet D, Alexander VJ, Baker BF, Brisson D, Tremblay K, Singleton W (2015). Antisense inhibition of apolipoprotein C-III in patients with hypertriglyceridemia. N Engl J Med.

[102] Digenio A, Dunbar RL, Alexander VJ, Hompesch M, Morrow L, Lee RG, Graham MJ, Hughes SG, Yu R, Singleton W, Baker BF, Bhanot S, Crooke RM (2016). Antisense-mediated lowering of plasma apolipoprotein C-III by volanesorsen improves dyslipidemia and insulin sensitivity in type 2 diabetes. Diabetes Care.

[103] Blom DJ, O'Dea L, Digenio A, Alexander VJ, Karwatowska-Prokopczuk E, Williams KR, Hemphill L, Muñiz-Grijalvo O, Santos RD, Baum S, Witztum JL (2018). Characterizing familial chylomicronemia syndrome: baseline data of the APPROACH study. J Clin Lipidol.

[104] Gelrud A, Digenio A, Alexander V, Williams K, Hsieh A, Gouni-Berthold I, Bruckert E, Stroes E, Geary R, Hughes S, Tsimikas S, Witztum J, Gaudet D (2018). Treatment with Volanesorsen (VLN) reduced triglycerides and pancreatitis in patients with FCS and sHTG vs placebo: results of the APPROACH and COMPASS. J Clin Lipidol.

[105] Warden BA, Duell PB (2018). Volanesorsen for treatment of patients with familial chylomicronemia syndrome. Drugs Today (Barc).

[106] Khetarpal SA, Zeng X, Millar JS, Vitali C, Somasundara AVH, Zanoni P (2017). A human APOC3 missense variant and monoclonal antibody accelerate apoC-III clearance and lower triglyceride-rich lipoprotein levels. Nat Med.

[107] Narayanan P, Shen L, Curtis BR, Bourdon MA, Nolan JP, Gupta S, Hoffmaster C, Zhou F, Christian B, Schaubhut JL, Greenlee S, Burel SA, Witztum JL, Engelhardt JA, Henry SP (2018). Investigation into the mechanism(s) that leads to platelet decreases in cynomolgus monkeys during administration of ISIS 104838, a 2′-MOE-modified antisense oligonucleotide. Toxicol Sci.

[108] Nikam RR, Gore KR (2018). Journey of siRNA: clinical developments and targeted delivery. Nucleic Acid Ther.

[109] Alexander VJ, Digenio A, Xia S, Hurh E, Hughes S, Geary RS, Witztum JL, Tsimikas S (2018). Inhibition of apolipoprotein C-III with GalNac conjugated antisense drug potently lowers fasting serum apolipoprotein C-III and triglyceride levels in healthy volunteers with elevated triglycerides. J Am Coll Cardiol.

